# Investigation of the Effect of Three Commercial Water Acidifiers on the Performance, Gut Health, and *Campylobacter jejuni* Colonization in Experimentally Challenged Broiler Chicks

**DOI:** 10.3390/ani13122037

**Published:** 2023-06-20

**Authors:** Tilemachos Mantzios, Vasilios Tsiouris, Georgios A. Papadopoulos, Vangelis Economou, Evanthia Petridou, Georgia D. Brellou, Ilias Giannenas, Costas G. Biliaderis, Konstantinos Kiskinis, Paschalis Fortomaris

**Affiliations:** 1 Unit of Avian Medicine, Clinic of Farm Animals, School of Veterinary Medicine, Aristotle University of Thessaloniki, 546 27 Thessaloniki, Greece; biltsiou@vet.auth.gr (V.T.); kiskinik@vet.auth.gr (K.K.); 2 Laboratory of Animal Husbandry, School of Veterinary Medicine, Aristotle University of Thessaloniki, 541 24 Thessaloniki, Greece; geopaps@vet.auth.gr; 3 Laboratory of Food Animal Hygiene and Veterinary Public Health, School of Veterinary Medicine, Aristotle University of Thessaloniki, 541 24 Thessaloniki, Greece; boikonom@vet.auth.gr; 4 Laboratory of Microbiology and Infectious Diseases, School of Veterinary Medicine, Aristotle University of Thessaloniki, 541 24 Thessaloniki, Greece; epetri@vet.auth.gr (E.P.); fortomap@vet.auth.gr (P.F.); 5 Laboratory of Pathology, School of Veterinary Medicine, Aristotle University of Thessaloniki, 546 27 Thessaloniki, Greece; mprellou@vet.auth.gr; 6 Laboratory of Nutrition, School of Veterinary Medicine, Aristotle University of Thessaloniki, 541 24 Thessaloniki, Greece; igiannenas@vet.auth.gr; 7 Department of Food Science and Technology, School of Agriculture, Aristotle University of Thessaloniki, 541 24 Thessaloniki, Greece; biliader@agro.auth.gr

**Keywords:** *C. jejuni*, water additives, poultry, broilers, One Health, water sanitation, biosecurity, gut health, toxicity

## Abstract

**Simple Summary:**

*Campylobacter jejuni* is considered the most common causative agent of human bacterial foodborne gastroenteritis worldwide with poultry products, and especially broilers meat, being the most common source. The aim of this study was to investigate the effect of the continuous application of three commercial water acidifiers (ProPhorce™ SA Exclusive, Premium acid, and Salgard^®^ Liquid) on the performance, gut health, and *C. jejuni* colonization in experimentally challenged broiler chicks. The experimental findings demonstrated that acidification of the drinking water by the tested products did not affect the transmission of *C. jejuni* from the seeder to sentinel birds, under the conditions used in this study. However, all the products ameliorated the adverse effects induced by *C. jejuni* infection in the physicochemical characteristics of the intestinal content. Finally, the continuous use of certain products from the first days of life evoked undesirable effects on broilers’ performance and induced severe gross lesions in the upper gastrointestinal tract, leading to the need to modify the dosage scheme in future investigations.

**Abstract:**

This study investigated the effect of three commercial water acidifiers on the performance, gut health, and *C. jejuni* colonization in experimentally challenged broiler chicks. A total of 192 one-day-old broiler chicks (Ross 308^®^) were randomly allocated into 6 treatment groups with 4 replicates according to the following experimental design: group A, birds were not challenged and received tap water; group B, birds were challenged and received tap water; groups C, D, E, and F, birds were challenged and received tap water treated with 0.1% *v*/*v* SPECTRON^®^, with 0.1–0.2% *v*/*v* ProPhorce™ SA Exclusive, with 0.1–0.2% *v*/*v* Premium acid, and with 0.1–0.2% *v*/*v* Salgard^®^ Liquid, respectively. The continuous water acidification evoked undesirable effects on broilers’ performance and to an increased number of birds with ulcers and erosions in the oral cavity and the upper esophageal area. ProPhorce™ SA Exclusive and Premium acid significantly reduced the *C. jejuni* counts in the crop, whereas Salgard^®^ Liquid significantly reduced the *C. jejuni* counts in the ceca of birds. At slaughter age, only Premium acid significantly reduced *C. jejuni* counts in the ceca of birds. All the tested products ameliorated the changes induced by *C. jejuni* infection in the pH in the ceca of birds. It can be concluded that besides the effectiveness of the tested products in controlling *C. jejuni* in broilers, their continuous application evoked undesirable effects on broilers’ performance, leading to the need to modify the dosage scheme in future investigations.

## 1. Introduction

*Campylobacter jejuni* (*C. jejuni*) is a gram-negative, microaerophilic bacterium that colonizes the gastrointestinal tract of various hosts (incl. monogastric species and ruminants) and can effectively survive in organic matter and environmental waters [1,2,3]. Since 2007, the microorganism has been recognized as the major cause of human bacterial foodborne gastroenteritis worldwide with the annual number of confirmed human cases in the European Union being approximately 130,000 for 2021 [4], resulting in an estimated cost of EUR 2.4 billion a year, due to the public health impact and lost productivity [5]. 

Poultry products, especially broiler meat, are considered the most important route for human infection by *C. jejuni*. Epidemiological surveys revealed that 60–80% of the analyzed poultry flocks are positive for this pathogenetic microorganism at slaughter age [6]. It is suggested that the naturally high body temperature of birds is favourable for the growth of the thermophilic *C. jejuni*, which colonizes birds’ gastrointestinal tracts, especially the region of the ceca, forming populations as high as 10^9^ CFU/gr of content [2]. Carcasses are subsequently contaminated at the slaughterhouse, especially during the evisceration process, which results in the augmentation of the pathogen in the food-production chain and, finally, its frequent isolation from retail poultry products [7].

The rise in annually reported human infections and the evermore frequent isolation of antimicrobial-resistant *C. jejuni* strains from humans, animals, and the environment [8,9] emphasize the urgent demand for the development of “alternative-to-antibiotics” biocides [10] and for the establishment of control strategies in line with the “One Health” approach [11]. Health organizations are showing an increased interest in alternative biocides, including organic acids, phytogenics, probiotics, and prebiotics. These substances have been found to have antimicrobial properties that can combat a variety of zoonotic pathogens. Additionally, they have positive effects on animal health, welfare, and productivity [12,13,14]. Overall, these compounds are deemed safe for both consumers and the environment and are in compliance with organic poultry production regulations [15,16]. 

According to the European Food Safety Authority (EFSA), Panel on Biological Hazards (BIOHAZ) 2020 for the update and review of the control options for *Campylobacter* spp., the addition of organic acids, chlorine-based biocides, or hydrogen peroxide to the drinking water of broilers could reduce the risk of *Campylobacter*-positive flocks up to 55% [13]. This recommendation is fully supported by previous epidemiological investigations in Great Britain, France, and Spain, which reveal that the sanitation of drinking water by organic or inorganic acids is strongly correlated with a lower percentage of *Campylobacter*-positive flocks [17,18,19].

Organic acids can act either as a source of carbon and energy for microorganisms or as inhibitory agents depending on the concentration of the acid, its ability to enter the cell, and the capacity of the organism to metabolize the acid [20]. The antibacterial action of organic acids involves several mechanisms. Organic acids lower the pH, creating an acidic environment hostile to bacteria. In addition, organic acids can penetrate the bacterial cell membrane and disrupt its integrity, leading to a loss of essential nutrients, or interfering with cellular metabolic processes, such as respiration and energy production, thus leading to the inhibition of specific enzymes and eventually to cell death [21].

Organic acids are traditionally used in livestock as preservatives or flavouring agents in feedstuff [20,21]. Moreover, it is reported that organic acids, such as butyric, formic, propionic, or acetic, could enhance growth, feed intake, feed efficiency, and egg production when applied to the feed or drinking water of poultry. In addition, organic acids have been linked with various physiological benefits, including immunological modulation and energy supply for intestinal cells [20,22]. Organic acids have also been reported to lower the pH of the gastrointestinal content of birds, creating a hostile microenvironment for the growth of pathogenic, pH-sensitive, bacteria [23] and thereby promoting the growth of beneficial microorganisms, such as *Lactobacillus* spp. and *Bifidobacterium* spp. Additionally, a reduction of the pH boosts the proteolytic activity of the gastric enzymes and, finally, enhances the digestion and absorption of nutrients [20,22,23]. 

The effect of water acidification on *C. jejuni* colonization in poultry has been investigated in only a few studies [24,25]. However, despite the promising findings from the in vitro and field data, in the in vivo experimental studies, no product was found to effectively prevent or reduce the counts in the ceca of birds to a degree that could reduce the incidence rate of human infection. In particular, Szott et al. [24] reported that adding the organic acids consistently reduced *C. jejuni* loads in cloacal swabs during the early stage of infection, whereas there were no significant differences in the *C. jejuni* loads of cecal and colon contents compared to the positive control group at the end of the study. Similarly, Mortada et al. [25], reported that an organic acid-based commercial product (consisting of formic acid and cinnamaldehyde) reduced the proliferation of *Campylobacter* spp. under the in vitro tests but it did not alter the *Campylobacter* spp. loads in the ceca of birds at 42 days of age. However, in a current systematic literature review of products with potential application for use in the control of *Campylobacter* spp. in organic and free-range broilers, Lassen et al. [26] concluded that a blend of organic acids could be a promising candidate for reducing *Campylobacter* spp. in broilers.

Thus, the aim of the present study was to investigate the effect of three commercial water acidifiers (ProPhorce™ SA Exclusive; Premium acid, and Salgard^®^ Liquid), which contain a blend of organic acids on the performance, gut health, and *C. jejuni* colonization in experimentally challenged broiler chicks.

## 2. Materials and Methods

### 2.1. Experimental Facilities, Biosecurity and Ethics

The experimental investigation was performed at the Aristotle University of Thessaloniki (AUTh), Greece, in the experimental facilities of the Unit of Avian Medicine, School of Veterinary Medicine. The Council Directive (2010/63/EU) and the Greek laws governing husbandry, euthanasia, experimental methods, and biosecurity precautions for experimental animals were followed and authorized by the Ethical Committee of the School of Veterinary Medicine and the Greek Veterinary Authority. 

Before the commencement of the animal study, all facilities and equipment were cleaned using a commercial alkaline foaming cleaning concentrate (Biosolve™ HD, Lanxess, Belgium) and disinfected by a commercial disinfectant (VIRAKIL^®^ NG, Ceva Santé Animale, France). Furthermore, two days before the experimentation, the drinking system was filled with a 3% solution of a water disinfectant based on hydrogen peroxide (Aqua-clean^®^, Kanters, Lieshout, The Netherlands) and left for 12 h, following a suitable flashing. Insecticide and rodenticide treatments were also performed in all experimental facilities and the surrounding environment. Finally, prior to the experiment, *C. jejuni* analysis was conducted to certify that the environment and the wood shavings that were used were *Campylobacter*-free. The temperature, the relative humidity, and the lighting were recorded (HOBO UX100-003 Temperature/Relative Humidity data logger, Onset Computer Corporation, Bourne, MA, USA) in each room on a daily basis, and they were adjusted following the recommendations of the breeding company (Aviagen^®^, Huntsville, AL, USA) [27]. Feed was also offered to the birds ad libitum throughout the trial. In order to stimulate commercial conditions, broilers were placed on fresh wood shavings, forming a litter of 5 cm in height.

### 2.2. Experimental Design 

#### 2.2.1. Experimental Groups

One hundred and ninety-two (192) one-day-old hatched broiler chicks (Ross 308^®^) were purchased from a local commercial hatchery. The birds originated from the same breeder flock and were vaccinated against infectious bursal disease (IBD) (CEVAC^®^ TRANSMUNE IBD, Ceva Animal Health Ltd., Libourne, France) by subcutaneous vaccination, and for Newcastle disease (ND) (AVINEW^®^, Boehringer Ingelheim Group, Ingelheim am Rhein, Germany) and infectious bronchitis (IB) (Cevac IBird, Ceva Animal Health Ltd., Libourne, France) by spray vaccination in the hatchery. After arrival, the birds were randomly allocated into 6 treatment groups with four replicates (8 chicks per replicate), summing a total of 32 chicks per group, according to the following experimental design: group A = birds were not challenged and received tap water without any treatment; group B = birds were challenged by *C. jejuni* and received tap water, without any treatment; group C = birds which were challenged by *C. jejuni* and received tap water treated with 0.1% *v*/*v* SPECTRON^®^ (0–15 d); group D = birds which were challenged by *C. jejuni* and received tap water treated with 0.1–0.2% *v*/*v* ProPhorce™ SA Exclusive; group E = birds which were challenged by *C. jejuni* and received tap water treated with 0.1–0.2% *v*/*v* Premium acid; and group F = birds which were challenged by *C. jejuni* and received tap water treated with 0.1–0.2% *v*/*v* Salgard^®^ Liquid. The entire duration of the experiment was 39 days, whereas the challenge of birds by *C. jejuni* was performed on the 18th day of age. All treatments were provided to birds as water additives, ad libitum, using 14 L tanks that were adjusted on the drinking line system and refilled when necessary (new solutions were prepared daily). 

#### 2.2.2. Feed

To meet the nutrient requirements of the broiler chicks, two complete basal diets were formulated for the starter (1 to 15 days) and the finishing period (16 to 39 d), respectively. No antibiotic growth promoters, organic acids, essential oils, or related anticoccidial drugs or/and mycotoxin binders were used. The feed synthesis and chemical analysis are shown in Table S1.

#### 2.2.3. Commercial Tested Products and Dosage Scheme

All the products used in this study are EU registered as water additives and are approved for use in poultry drinking water. The synthesis of the selected commercial products and the manufacturer’s recommendations are presented in Table 1.

**Figure 1:**
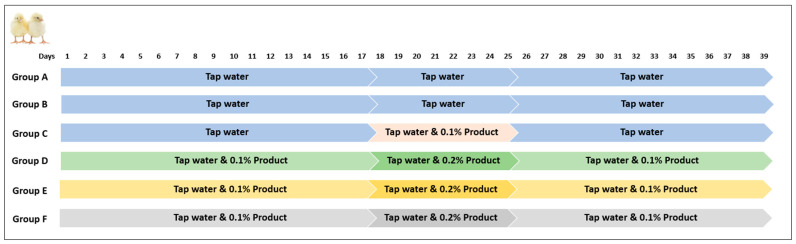
Tested products dosage scheme [group A = birds were not challenged and received tap water without any treatment; group B = birds were challenged by *C. jejuni* and received tap water without any treatment; group C = birds which were challenged by *C. jejuni* and received tap water (0–15 d) treated with 0.1% *v*/*v* SPECTRON^®^; group D = birds which were challenged by *C. jejuni* and received tap water treated with 0.1–0.2% *v*/*v* ProPhorce™ SA Exclusive; group E = birds which were challenged by *C. jejuni* and received tap water treated with 0.1–0.2% *v*/*v* Premium acid and group F = birds which were challenged by *C. jejuni* and received tap water treated with 0.1–0.2% *v*/*v* Salgard^®^ Liquid].

As a water supply, a 14 Lt container (tank) was filled daily with only fresh tap water or water combined with the products used for treatments, as listed in Table 1 and provided to birds through the drinking line. Commercial water acidifiers were applied to the birds immediately after their arrival on the first day according to the treatment-dosage scheme illustrated in Figure 1. After the preparation of the treatment solution, water samples (50 mL) were collected daily from each group, and the pH was determined using a digital pH meter (pH 315i, WTW Wissenschaftlich-Technische Werkstatten, Weilheim, Germany). Chemical and microbiological analysis of the tap water was also performed at the beginning of the trial.

### 2.3. Mycotoxin Analysis in Feed

Samples were collected for mycotoxin analysis from both starter and finisher diets, as previously described by Tsiouris et al. [28]. Feed samples were analyzed by LC/MS-MS for fumonisins detection [29] and the rest of the mycotoxins were analysed as described by Tassis et al. [30]. The results were recorded as the detected mycotoxins levels/detection limit level.

### 2.4. Campylobacter jejuni Strain, Inoculum Preparation, Birds’ Infection 

The *C. jejuni* strain MB 4185 KC 40 was kindly provided by Professor Frank Pasmans (Faculty of Veterinary Medicine, Ghent University, Merelbeke, Belgium). This strain has been used in previous investigations [31,32,33] and was highly efficient in colonizing broilers by forming large populations in the gastrointestinal tract [33]. The strain was stored at −80 °C in a 40% glycerol solution enriched with 5% defibrinated horse blood. After restoration, 100 μL of the initial suspension were plated on 5% horse-blood agar plates (Columbia horse-blood agar plates, Bioprepare). Plates were enclosed in airtight jars (Genbox jar, Biomérieux, Marcy-l’Étoile, France) and incubated under a microaerobic atmosphere (Thermo Scientific™ Oxoid™ CampyGen™ 2.5 L Sachet, Oxoid, Basingstoke, UK), at 42 °C, for 48 h. Following this, a second culture (working culture) was prepared. Colonies from the working culture were dispersed with a sterile loop in buffered peptone water (PBW, Oxoid, Basingstoke, UK) to obtain a turbidity of 0.5 McFarland. Finally, birds were challenged by an oral gavage of 1 mL PBS containing approximately 6.5 × 10^6^ CFU/mL *C. jejuni*. The concentration of *C. jejuni* in the final inoculum was determined by plate counting on 5% horse-blood agar plates (Columbia horse-blood agar plates, Bioprepare, Athens, Greece) of decimal dilutions and incubation under appropriate conditions. Results were obtained two days after the challenge of the birds. 

**Figure 2:**
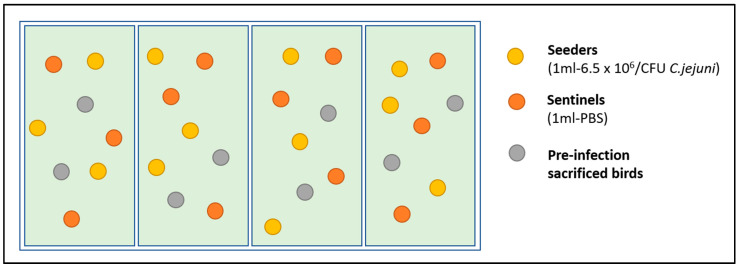
Illustrative scheme of the seeder-bird model that was applied for the investigation of the effect of the commercial water acidifiers in the control of *C. jejuni* in experimentally challenged broilers.

A stochastic seeder-bird model was applied as previously reported [24] in order to investigate the effect of the commercial water acidifiers in the control of *C. jejuni* in broilers and, thus, the birds in each replicate were divided into three categories (Figure 2) and were marked by a unique sequential number (individual wing tagging). In each replicate*, broilers were assigned as:

- “pre-infection sacrificed birds” (*n* = 2): birds were not challenged, as they were sacrificed on the 15th day of age;
- “seeders” (*n* = 3): birds were challenged at the 18th day of age, by oral gavage of 1 mL PBS containing 6.5 × 10^6^ CFU *C. jejuni;*
- “sentinels” (*n* = 3): birds were not challenged, but received orally 1 mL of sterile PBS as a “*placebo*”

*In the negative control group, all the birds in each of the replicates received only sterile PBS as a “*placebo*”.

### 2.5. Records

#### 2.5.1. Performance Evaluation

During this trial, clinical signs and/or mortality were recorded daily. The body weight (BW) of birds was measured on the 1st, 9th, 15th, 20th, 23rd, 25th, 30th, 37th, and 39th day of age, while the average daily feed intake (ADFI), the average daily weight gain (ADWG), and the feed conversion ratio (FCR) were calculated for the periods of the 1st–9th day, 1st–15th, 16th–39th, and 1st–39th day. The European production efficacy factor (EPEF) was also calculated for the total experimental period by taking into account the final body weight and the livability percentage (%). Additionally, the water intake (WI) per group was calculated by recording daily (at 8:30 a.m.) the remaining water in the tank and subtracting it from the water volume that was prepared the day before. However, the drinking line system utilized in our study had the same tank shared between replicates, which restricted our ability to record daily water consumption on an individual replicate basis. Consequently, the recorded data for water consumption was aggregated at the group level and could not be evaluated statistically due to the absence of statistical replicates. 

#### 2.5.2. Gross Lesion Score

At the 39th day of age, during sampling, the gastrointestinal tract and the liver (*n* = 12/group) were macroscopically examined and scored for gross lesions. In particular, the intestines were macroscopically examined and scored with a scale ranging from 0 to 4 for coccidiosis [34]. The coccidiosis score was evaluated for each *Eimeria* strain: *E. acervulina* in the duodenum, *E. maxima* in the duodenum/jejunum, and *E. tenella* in the ceca. Additionally, the intestines were macroscopically examined and scored for dysbacteriosis lesions, as previously described by Teirlynck et al. [35]. In particular, the dysbacteriosis lesion scoring system entailed a 10-point categorical scale and each bird was given a score between 0 and 10, where 0 reflects a normal gastrointestinal tract and 10 where the most severe dysbacteriosis lesions occurred. Parameters such as gut ballooning, inflammation, thickness, undigested feed, etc. were assessed, giving each one a score of 0 when it was absent and 1 when was occurred. Finally, the overall dysbacteriosis score was addressed by summing each lesion’s individual score. The livers were macroscopically examined and scored for gross lesions using a 0–2 scale, as described by Tsiouris et al. [36], giving a score of 0 when no gross lesions were observed, a score of 1 when liver congestion and/or gallbladder distention and wall thickening and/or bile discoloration were observed, and a score 2, when necrotic lesions in the liver were observed. The gizzards were macroscopically examined and scored for gross lesions using a 0–2 scale, as described by Novoa-Garrido et al. [37]. Finally, a 0–1 (yes/no) scale lesion-scoring system was performed for the upper gastrointestinal tract of birds (oral cavity and esophagus), giving a score of 1 in birds where gross lesions (erosions or ulcers) were presented, while a score of 0 was given to birds with a normal epithelial surface. 

#### 2.5.3. Histopathological Analysis

When gross lesions (erosions and/or ulcers) were observed in the upper gastrointestinal tract (oral cavity/esophagus), histological samples were collected, fixed in 10% neutral-buffered formalin for 48–72 h and processed routinely. Finally, 3–5 μm thick sections were stained with hematoxylin and eosin (H–E) and evaluated using an optical microscope.

#### 2.5.4. Physicochemical Measurements of Intestinal Content

At the 15th and 39th day of age after euthanasia, the content of the crop, gizzard, duodenum, jejunum, ileum, and ceca (15th day: *n* = 8/group; 39th day: *n* = 12/group) was immediately collected in separate tubes (12 mL) and vortexed individually in order to obtain a homogeneous content from each anatomical part of the gastrointestinal tract. The pH was measured using a digital pH meter. The crop and stomach were opened during the necropsy process and the pH was directly measured using the digital pH meter. 

Additionally, the homogenized contents from the jejunum and the ileum of the sampled birds (15th day: *n* = 8/group; 39th day: *n* = 12/group) were collected in separate tubes (12 mL) and examined for viscoelastic assay, as previously described by Tsiouris et al. [28]. Shortly, the tubes with homogeneous content from each anatomical part of the intestine were centrifuged at 3000× *g* for 15 min to separate the feed particles from the liquid phase. Supernatants (1 mL) from each tube were collected, and the viscosity was measured in a rotational Physica MCR 300 rheometer (Physica Messtechnic GmbH, Stuttgart, Germany). The data of the rheological measurements were analyzed with the supporting rheometer software US200 V2 and represented in centipoise (cP) units. 

#### 2.5.5. Microbiological Analysis

##### *C. jejuni* Enumeration

At the 25th (7 days post-infection) and 39th day (21 days post-infection) of age, the crop and the ceca (15th day: *n* = 12/group; 39th day: *n*_crop_ = 6/group, *n*_ceca_ = 12/group) were aseptically collected in sterile stomacher bags and immediately transported for enumeration of *C. jejuni*. The samples were examined with the ISO 10272-2:2006 method with modifications. In brief, crop and cecal samples were weighed and put into a sterile stomacher bag containing a nine-fold volume of Maximum Recovery Diluent (MRD, Oxoid, Basingstoke, UK), and the blend was homogenized for 60 s by Stomacher. From the initial, decimal dilutions were performed in MRD-containing tubes. From every dilution, surface platting was performed in charcoal cefoperazone deoxycholate agar (mCCDA 10409; Liofilchem^®^ S.r.l, Roseto degli Abruzzi, Italy) supplemented by CCDA selective supplement (81037; Liofilchem^®^ S.r.l, Roseto degli Abruzzi, Italy). Plates were then placed into sealed jars and incubated for 48 h at 42 °C under microaerophilic conditions (85% N_2_, 10% CO_2_, 5% O_2_; Thermo Scientific™ Oxoid™ CampyGen™ 2.5 L Sachet, Oxoid, Basingstoke, UK). 

After incubation, presumptive positive *C. jejuni* colonies were counted and confirmed by standard biochemical and molecular methods, including peroxidase reaction, direct microscopy, gram staining, and PCR. Results were demonstrated as total *C. jejuni* CFU/g of the sample and expressed as log_10_ CFU/g of the ceca for further statistical analysis.

The PCR method was performed as previously described by Tsiouris et al. [32] with modifications. DNA extraction was performed by dispersing one pure culture in 100 μL dispersal buffer. The composition of the PCR reagents used is shown in Table S2. Subsequently, 100 μL of lysis buffer I and 25 μL of proteinase K solution (22.4 mg/mL) were added. After incubation at 56 °C for 1 h, 250 μL of lysis buffer II were added and further incubated at 70 °C for 10 min. A volume of 250 μL of absolute ethanol were added and the mixture was passed through silica columns (Qiagen Purification Technologies, Hilden, Germany). The column was then washed (2× wash I buffer and 1× wash buffer II) and the DNA was eluted with ultrapure water and stored at −30 °C until examination. Two primer sets were used [38] for the detection of *C. jejuni* hipO (CJF: ACT TCT TTA TTG CTT GCT GC, CJR: GCC ACA ACA AGT AAA GAA GC; product 323 bp) and 23S rRNA genes (23SF: TAT ACC GGT AAG GAG TGC TGG AG, 23SR: ATC AAT TAA CCT TCG AGC ACC G; product 650 bp). The PCR was performed at a volume of 25 μL containing 200 μM dNTPs, 2 mM MgCl_2_, 1 μM CJF/CJR primers, 0.2 μM 23SF/23SR, and 1.25 U Taq polymerase. All PCR reagents were purchased from New England Biolabs. After initial denaturation for 6 min at 95 °C, 30 cycles of amplification were performed (denaturation 95 °C/30 s, annealing 59 °C/30 s, and extension 72 °C/30 s), with a final extension at 72 °C for 7 min. The PCR products were visualized in 1.5% agarose gels stained with ethidium bromide. 

##### *Cecal microbiota* Enumeration

At the 15th and 39th day of age, ceca (*n* = 8/group) were collected during samplings, in order to determine the counts of essential bacteria commodities such as *E. coli*, *Clostridium* spp., *Lactobacillus* spp., and *Bifidobacterium* spp., as previously described by Tsiouris et al. [34], and according to the ISO16649-2: 2001, ISO7937:2004, ISO 15214:1998, and ISO29981:2010, respectively.

### 2.6. Statistical Analysis

The effect of the challenge by *C. jejuni* and the treatment of the drinking water by the tested products were analyzed with one-way ANOVA using SPSS 28.0 for each time point (BM SPSS Statistics for Windows, Version 28.0. Armonk, NY, USA: IBM Corp.). Post hoc comparisons between treatments were investigated by the Duncan test. The average values including the standard error of the mean (SEM) were calculated for every examined parameter and presented in the tables. Statistical analysis of the scores (gizzard score, liver score, dysbacteriosis score, and coccidiosis lesion score in the intestine) was performed by Chi-squared tests applied with SPSS as well as with the use of GraphPad Prism (version 9.1.2 for Windows^®^, GraphPad Software, San Diego, CA, USA). The statistical unit for the evaluation of FCR, ADWG, ADFI, and EPEF was the pen (replicate), while the statistical unit for the other analysis (BW, intestinal pH and viscosity, results from microbiology, and gross lesion scores) was the bird. The level of significance was set at *p* ≤ 0.05 and statistically significant trends (#) were underlined for *p* ≤ 0.1.

## 3. Results

### 3.1. Performance Evaluation

Throughout the entire period (1–39 days), mortality was not recorded. Daily clinical examination revealed sporadic clinical signs in the challenged groups, such as diarrhoea, ruffled feathers, and growth retardation. The effects of the *C. jejuni* challenge, and of the different experimental treatments on the performance of birds in different periods of the experiment, are presented in Table 2 and Table 3.

The challenge by *C. jejuni* had no significant (*p* > 0.05) impact on the BW of birds during the experimental period. However, at slaughter age, the final mean BW of birds in the positive control group (group B: 2532 g) was numerically lower, by 100 g, compared to that in the negative control group (group A: 2634 g). As early as the 15th day of age, the BW of birds in groups D and E was lower (*p* ≤ 0.05) compared to the controls (groups A, B, C). While the BW of birds in group A did not significantly (*p* > 0.05) differ from those of group B (after the challenge, 18th day of age), the BW of birds in groups D and E was a lower (*p* ≤ 0.05) compared to those of group A at 20th, 23rd, and 25th day of age. Finally, at slaughter age, the mean BW of birds was not significantly (*p* > 0.05) different among the experimental groups; however, a statistical trend (*p* = 0.08) was recorded for the mean BW of birds in group D, being significantly lower in comparison with that in groups A and F.

**Table 3:** Effect of drinking water acidification on the performance [FCR, ADWG, ADFI, and EPEF ($\bar{x}$ ± SEM)] of broiler chicks, experimentally challenged by *C. jejuni*.

Parameter	Period	Experimental Groups						*p* Value
		A	B	C	D	E	F	
FCR (g:g)	1–9 d *	1.24 ± 0.01			1.29 ± 0.02	1.30 ± 0.02	1.26 ± 0.02	0.075
	1–15 d *	1.50 ± 0.02			1.47 ± 0.05	1.48 ± 0.02	1.42 ± 0.02	0.245
	15–39 d	1.56 ± 0.03	1.57 ± 0.01	1.63 ± 0.01	1.68 ± 0.07	1.62 ± 0.03	1.55 ± 0.01	0.116
	1–39 d	1.56 ± 0.04	1.55 ± 0.02	1.59 ± 0.02	1.61 ± 0.06	1.58 ± 0.02	1.51 ± 0.01	0.374
ADWG (g)	1–9 d *	20.83 ± 0.45			20.30 ± 0.56	20.08 ± 0.42	20.10 ± 0.84	0.714
	1–15 d *	36.40 ± 0.91			34.29 ± 0.70	34.33 ± 0.97	35.36 ± 1.95	0.500
	15–39 d	91.40 ± 1.38	87.78 ± 4.67	84.71 ± 3.37	78.09 ± 3.12	84.44 ± 0.81	89.06 ± 3.53	0.094
	1–39 d	68.12 ± 0.90	65.43 ± 3.11	63.53 ± 2.20	59.01 ± 1.93	62.98 ± 0.45	66.06 ± 2.12	0.071
ADFI (g)	1–9 d *	26.40 ± 0.46			26.58 ± 0.55	26.54 ± 0.62	25.63 ± 0.74	0.416
	1–15 d *	40.75 ± 0.43 ^b^			38.37 ± 1.05 ^a^	38.31 ± 0.60 ^a^	37.29 ± 0.75 ^a^	0.003
	15–39 d	134.21 ± 1.04	126.72 ± 4.59	129.52 ± 2.66	124.47 ± 5.23	126.49 ± 0.95	129.08 ± 2.64	0.410
	1–39 d	86.75 ± 2.02 ^b#^	80.41 ± 2.43 ^a#^	81.40 ± 1.30 ^a,b#^	78.11 ± 2.85 ^a#^	79.01 ± 0.50 ^a#^	79.66 ± 1.32 ^a#^	0.060
EPEF	1–39 d	437.79 ± 16.30 ^b^	421.21 ± 19.98 ^b^	400.30 ± 16.41 ^a,b^	367.19 ± 19.40 ^a^	398.77 ± 5.37 ^a,b^	437.23 ± 15.43 ^b^	0.048

^a,b^ Means in the same row with a different superscript differ significantly (*p* ≤ 0.05). ^#^ Means in the same row with a different superscript differ significantly (*p* ≤ 0.10). * For the period 1–18 days, groups A, B, and C were considered as one (controls), as birds in these groups received only tap water (without any treatment), and the challenge by *C. jejuni* was not yet applied. FCR: Feed conversion ratio; ADWG: Average daily weight gain; ADFI: Average daily feed intake; EPEF: European production efficiency factor.

The challenge by *C. jejuni*, as well as its combination with the water treatments, had no significant (*p* > 0.05) impact on the FCR and the ADWG of birds during the experimental period. Instead, the ADFI for the period of 1–15 days was lower (*p* ≤ 0.05) in groups D, E, and F compared to that of the controls (groups A, B, and C). Additionally, for the total experimental period (1–39 days), the ADFI in groups B, D, E, and F trended to be significantly (*p* = 0.06) lower compared to group A. Finally, the calculation and the statistical analysis of the EPEF (each replicate was a statistical unit) revealed that group D resulted in significantly (*p* ≤ 0.05) lower EPEF (1–39 days) compared to groups A, B, and F.

**Figure 3:**
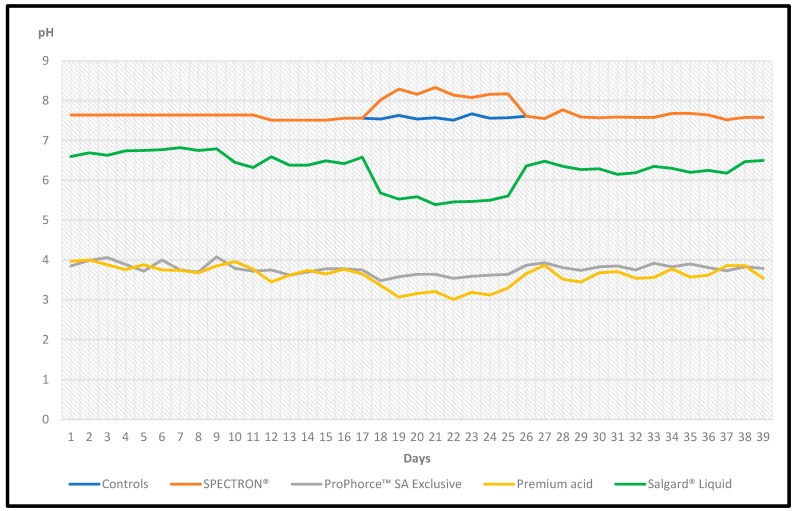
Effect of the tested products on the pH of the drinking water (daily measurements) during the entire experimental period.

The chemical analysis of the nonmedicated tap water (control), as well as the effect of the treatments on its pH throughout the entire experimental period, is represented in Table 4 and Table 5, and in Figure 3.

The application of all the products altered significantly (*p* ≤ 0.05) the pH of the drinking water compared to that of the nonmedicated tap water. In particular, the application of ProPhorce™ SA Exclusive in the concentration of 0.1% *v*/*v* reduced significantly (*p* ≤ 0.05) the pH of the drinking water by 3.78 units, whereas when applied in 0.02% *v*/*v* reduced it by 3.98 (*p* ≤ 0.05). Similarly, the application of Premium acid in the concentration of 0.1% *v*/*v* reduced significantly (*p* ≤ 0.05) the pH of the drinking water by 3.88 units, whereas when applied at 0.02% *v*/*v* reduced it by 4.39 (*p* ≤ 0.05). Salgard^®^ Liquid in the concentration of 0.1% *v*/*v* significantly (*p* ≤ 0.05) reduced the pH of the drinking water by 1.13 units, whereas when applied at 0.02% *v*/*v* reduced it by 2.04 (*p* ≤ 0.05). Finally, the application of 0.1% *v*/*v* Spectron^®^ significantly (*p* ≤ 0.05) increased the pH of the drinking water by 0.6 units.

The effect of the water acidification and *C. jejuni* challenge on the water intake (WI), as well as its ratio with feed intake (WI/FI), is presented in Table 6. 

### 3.2. Gross Lesion Score

The effect of the *C. jejuni* challenge and its combination with the tested water treatments on the dysbacteriosis lesion score, which was performed in the intestine of broilers at the 39th day of age, is presented in Table 7. 

**Figure 4:**
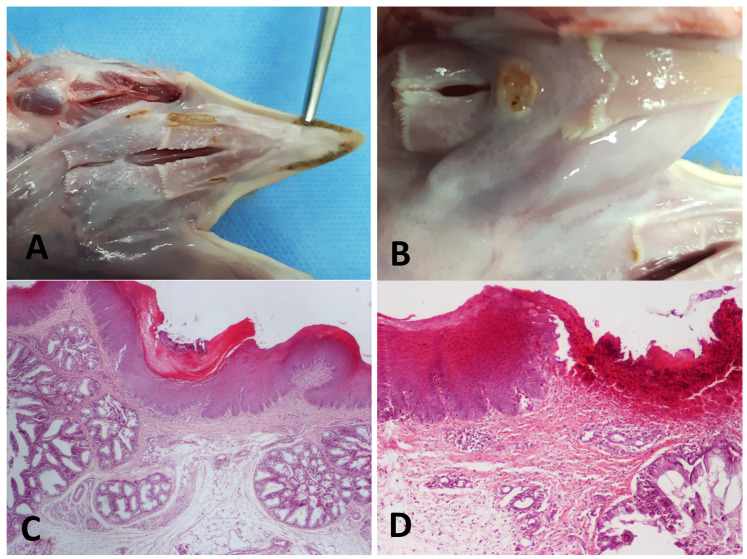
Images from the gross and histopathological lesions that were recorded in the oral cavity or/and the esophagus of 15-day-old broiler chicks that received acidified water. (**A**) Palate: three small necrotic foci and one larger and fusiform are obvious. The latter, located parallel to the choana, has a coarse surface and is delineated by a yellowish halo. (**B**) An ovoid to round-shaped ulcer located at the root of the tongue, rostrally situated to the glottis, is also seen. (**C**) Histopathological section depicting an oral mucosal erosion [H–E, original magnification × 10]. (**D**) Histopathological section depicting an esophageal ulcer [H–E, original magnification × 10].

No lesion was recorded according to the coccidiosis lesion score, foot-pad lesion score, gizzard, and liver score. However, at the 15th day of age during necropsy, gross lesions in the oral cavity and the esophagus (Figure 4A,B) of birds were recorded, and, thus, samples were randomly collected for further histopathological investigation. The percentage (%) of birds with gross lesions on the upper gastrointestinal tract is presented in Table 8.

The percentage of birds with gross lesions in the oral cavity and/or esophagus in groups D and E has significantly (*p* ≤ 0.05) increased compared to that in the control groups (groups B and C). In addition, the number of birds with gross lesions was significantly (*p* ≤ 0.05) lower in group F compared to that in group D.

### 3.3. Histopathological Findings

Samples from the oral cavity and esophagus were collected, based on rare macroscopic lesions that were noted during sampling on the 15th day of age. Histopathological lesions mainly included erosions and in some cases ulceration of oral and esophageal mucosa (Figure 4C,D). In most cases, hyperkeratosis was a common finding, while vacuolar degeneration and the presence of vesicles were rarely observed.

### 3.4. Physicochemical Characteristics of the Intestinal Content

Data on the pH of the contents in the various anatomical parts of the gastrointestinal system of birds, on the 15th and 39th days of age, are presented in Table 9.

On the 15th day, the pH values of the contents in the crop, gizzard, duodenum, jejunum, and ceca were not significantly (*p* > 0.05) different among the experimental groups. However, the pH of digesta in the ileum of birds in group D significantly (*p* ≤ 0.05) increased compared to that of birds in the control groups (A, B and C), as well as to that of groups E and F. Neither the challenge by *C. jejuni* nor its combination by antibiotic treatment, or the water acidifiers had a significant (*p* > 0.05) impact on the pH of the contents from crop, duodenum, jejunum, and ileum of birds at 39 days of age. However, at slaughter age, the pH in the ceca of birds in group B (positive control) was significantly higher (*p* ≤ 0.05) compared to the others.

Data of viscosity measurements made on the intestinal contents in the jejunum and ileum parts of birds, on the 15th and 39th days of age, are presented in Table 10. At the 15th day of age, the viscosity of the contents in the ileum and jejunum of birds did not significantly (*p* > 0.05) differ among the experimental groups. However, at the 39th day of age (21 days post-infection), the viscosity of the intestinal contents in the jejunum of birds in group B has significantly (*p* ≤ 0.05) increased, compared to that in groups A, E and F.

### 3.5. Microbiological Analysis

#### 3.5.1. *C. jejuni* Enumeration

The effect of the tested water treatments on the *C. jejuni* counts in the crop and the ceca of broiler chicks at the 25th and 39th day of age is presented in Table 11.

*C. jejuni* was not detected in the crop and the ceca of birds in group A (the negative control), highlighting the efficiency of the restricted biosecurity measures that were applied during experimentation. Neither the Spectron^®^ nor the tested water acidifiers reduced the dynamics of the *C. jejuni* transmission among birds, as the bacterium was detected in all the sentinel birds that were sampled seven days post-infection (transmission rate of 100%) in all the challenged groups. On the 25th day of age (7 days post-infection), the *C. jejuni* counts in the crop of birds in groups C, D, and E were lower (*p* ≤ 0.05) compared to group B. Additionally, *C. jejuni* counts in the ceca of birds in groups C and F were also lower (*p* ≤ 0.05) compared to group B, while the counts in group C were lower (*p* ≤ 0.05) than those of groups D and E. At the 39th day of age (21 days post-infection), *C. jejuni* counts in the crop did not differ (*p* > 0.05) among the experimental groups. Instead, the *C. jejuni* counts in the ceca of groups C and E were found lower (*p* ≤ 0.05) than those in group B.

#### 3.5.2. Cecal Microbiota Enumeration

The effects of the *C. jejuni* challenge and its combination with the tested water treatments in the composition of the cecal microbiota in broilers, at the 15th and 39th days of age, are shown in Table 12.

At the 15th day of age (3 days pre-challenge), none of the water acidifiers induced significant (*p* > 0.05) alterations in the cecal microbiota composition. However, in group F, the counts of *Clostridium* spp. in the ceca seemed to be lower (*p* = 0.058) compared to those in groups A, B, and D. Similarly, at the 39th day of age, neither the challenge nor its combination with the water treatments altered significantly (*p* > 0.05) the counts of *E. coli*, *Lactobacillus* spp., and *Bifidobacterium* spp. in the ceca of birds. However, the counts of *Clostridium* spp. in all the challenged groups (B, C, D, E, and F) have increased (*p* ≤ 0.05) significantly (by 1.70, 1.63, 1.78, 1.12, and 1.72 log_10_ CFU/g, respectively) compared to the negative control group (group A).

## 4. Discussion

This study investigated the effect of three commercial water acidifiers (ProPhorce™ SA Exclusive, Premium acid, and Salgard^®^ Liquid) on the performance, gut health, and *C. jejuni* colonization in broiler chicks which were experimentally challenged by the *C. jejuni*. Our results demonstrated that continuous acidification of the drinking water did not affect the transmission of *C. jejuni* from the seeder to sentinel birds, under the conditions used in this study. In addition, the continuous use of certain products from the first days of life evoked undesirable effects on the broilers’ performance, leading to the need for modifying the dosage scheme in future investigations.

Several factors can affect the pathogenicity of *C. jejuni* in poultry, including the bird’s genetic background, overall health, management, strain type, infectious dosage, and age of infection [39,40]. These factors can cause variations among experimental studies and finally make it difficult to determine whether *C. jejuni* is a pathogen or a commensal bacterium for poultry [41]. In the present study, birds were infected at the 18th day of age by oral ingestion of 1 mL of 10^6^ CFU of *C. jejuni* reference strain KC40. In both samplings, 7- and 21-days post-infection, *C. jejuni* was detected in the ceca of infected birds in populations of ∼7 log_10_ CFU/g and ∼8 log_10_ CFU/g content (Table 11), which is in accordance with previous investigators demonstrating that the *C. jejuni* K40 is efficient in colonizing chickens to a high level [32,33,42].

Certain *C. jejuni* strains, such as INN-1-179, C101, ATCC 33291, and NCTC 12744, have been previously reported to adversely affect the performance of broilers in terms of reduced ADFI and ADWG [39]. Infection by *C. jejuni* K40 did not significantly alter the performance parameters in this study. However, at slaughter age, the final mean BW of the only challenged birds was numerically lower, by 100 g, compared to that of the nonchallenged birds. In addition, a statistical trend for lower ADFI over the total experimental period was recorded in the challenged birds, compared to their nonchallenged counterparts. Previous investigators demonstrated that infection of broilers by *C. jejuni* can lead to decreased growth rates due to poor nutrient absorption in the intestines, changes in the structure of the gut, shifts in the gut microbiota, and abnormal behaviour in affected birds [39,43,44]. 

It is reported that *C. jejuni* colonization may induce shifts in the composition of the gut microbiome of the infected birds by affecting the development and complexity of the microbial communities [39]. In some previous studies, infection of broilers by *C. jejuni* resulted in an increased abundance of *Clostridium* spp. and a decreased abundance of *E. coli* in multiple areas of the intestine [45,46]. In agreement with the above studies, infection of broilers by *C. jejuni* KC40 in the present study increased *C. perfringens* counts in the ceca of birds at the 38th day of age. It is suggested that *C. jejuni* acts as a hydrogen sink, which enhances the growth of *Clostridia* through increased fermentation and organic acid production [47], which *C. jejuni* can also utilize as an energy source. 

*C. jejuni* infection in this study significantly increased the pH of intestinal contents in the ceca of birds. It is reported that *C. jejuni* infection modulates the profile of metabolic end products derived from the intestinal microbiota in broilers. Awad et al. [48] conducted a study that revealed how *C. jejuni* infection lowers the amount of certain SCFAs in the intestine of the infected birds, suggesting that the metabolic activity of some bacteria in the gut is altered. As a result, the pH levels in the jejunum and caecum digesta of infected birds in the above study increased to a level that is ideal for the growth of *C. jejuni*.

The viscosity of the intestinal digest is mainly affected by the presence of water-soluble non-starch polysaccharides (NSP), which are mostly found in cereal grain, as well as by the ability of goblet cells to produce mucin 2, a glycoprotein that is crucial for intestinal epithelial mucus-layer formation [49,50,51]. Higher intestinal viscosity has been associated with poor gut health and broiler performance [52]. The higher viscosity of the intestinal content leads to the increased retention time of the digesta, which allows more time for the pathogens to colonize the gastrointestinal tract. The higher viscosity also reduces conjugated bile acids, affecting fat emulsification and digestibility [51], while simultaneously increasing the gut passage time and, therefore, the amount of undigested materials in the intestines of birds. 

Previous research has shown that changes in the viscosity of the intestinal digesta are strongly associated with intestinal diseases such as necrotic enteritis and coccidiosis [53]. For *C. jejuni*, such a relationship still needs to be elucidated. In the study of Fernandez et al. [54], lower *C. jejuni* numbers were associated with lower jejunal viscosity. Additionally, Molnár et al. [55] found that *C. jejuni* infection induces excess mucus production in the intestine, as indicated by the higher viscosity of the intestinal digest of infected birds. While the mucus layer in the intestine of birds represents a significant barrier for the attachment and invasion of several bacteria into the epithelial cells [50], it seems that, for *C. jejuni*, this mechanism is not applicable. Actually, the presence of the flagella and the darting motility of *C. jejuni* are important advantages, facilitating the movement of this bacterium in a highly viscous environment such as intestinal mucus [56]. In addition, the microaerophilic environment and the nutrients in the intestinal mucus seem to enhance the growth and adhesion of *C. jejuni* to the epithelial cells. 

In the present study, infection of broilers by *C. jejuni* did not significantly affect the viscosity of the contents in various anatomical parts of the birds. However, at the 38th day of age (21 days post-infection), the viscosity in the positive control group was numerically lower compared to that of the negative control. This could be attributed to the osmotic and absorptive alterations induced by the *C. jejuni* infection in the birds’ gastrointestinal tract [57,58]. Moreover, reduced viscosity could be associated with watery diarrhoea observed during the clinical examination of some infected birds in this study. 

In this study, water acidifiers were applied in a continuous dosage scheme to investigate their efficiency in controlling *C. jejuni* colonization dynamics. It is suggested that broilers receiving acidified water from the first days of their life would be less susceptible to colonization by enteric pathogens after exposure, due to alterations in the host gut microenvironment and the host immune system [59]. However, various parameters, including the type of acidic agents employed, dosage scheme, water quality, feed buffering capacity, feed ingredients, host microbiota composition, and general health of birds, as well as general farm management, might all have an impact on the efficacy of commercial formulas and, thereby, occasionally lead to unfavourable outcomes [60,61,62,63]. 

In the present study, the continuous water acidification by both ProPhorce™ SA Exclusive and Premium acid has resulted in the desired augmentation in the mean BW of birds, as early as the 15th day of age. In addition, while *C. jejuni* challenge did not significantly affect the BW of birds at any timepoint of this trial, its combination with ProPhorce™ SA Exclusive or Premium acid resulted in significantly lower BW of birds on the 20th, 23rd, and 25th days, compared to the negative control group. Our results are in agreement with previous studies that also reported adverse effects on the performance of birds being subjected to a continuous water acidification program [61,62,63].

It is reported that the overuse of organic acids in the drinking water of birds may cause lower water consumption and, subsequently, lower feed intake due to changes in the odour and taste of the drinking water [62,63]. Generally, birds prefer water to be slightly acidic [64]. According to the broiler management guide of Aviagen^®^, the preferred pH for optimal performance is between 5.0–6.0 [27]. pH values greater than 8.0 may cause unsatisfactory bacterial growth and biofilm formation, whereas pH values lower than 4.0 may result in poor performance, damage to the equipment, and fungal overgrowth [27]. Açıkgöz et al. [63] reported that the continuous addition of formic acid in the drinking water of broilers resulted in a pH of 4.5 that significantly decreased the BW of birds at the 21st and 42nd day of age. In the present study, both products are highly concentrated in formic acid, whereas their application at the tested dosages induced an extremely low pH below 4.0 (3.18–3.82). However, water intake could not be evaluated statistically due to the absence of replicates. Nevertheless, no remarkable fluctuations in water consumption as a whole were recorded among the experimental groups (Table 6). 

On the other hand, the ADFI for the period 1–15 days was reduced in the groups where water acidifiers (ProPhorce™ SA Exclusive, Premium acid, and Salgard^®^ Liquid) were applied continuously at the concentration of 0.1% *v*/*v*. On the 15th day of age, the post mortem examination revealed that in the groups where the drinking water was treated by ProPhorce™ SA Exclusive and Premium acid, gross lesions (erosions and/or ulcers) in the oral cavity and/or esophagus (Figure 4A,B) were recorded in a higher percentage (*p* ≤ 0.05) of birds (Table 8). Similar lesions can be caused by foreign-body injury, toxic ingestion (including mycotoxins), parasitic infections (e.g., *Trichomonas gallinae*), or due to treatment with ulcerogenic drugs [65,66,67,68]. The feed analysis ruled out mycotoxins as the cause of the lesions observed. Moreover, the increased biosecurity applied in the experimental facilities and the quality of the feed ingredients could not support the association between the recorded lesions and foreign items or other toxic substances. Additionally, the histopathological findings were not compatible with parasitic or fungal infection (Figure 4C,D). The ulcers and erosions found in this study appear to be strongly connected to the drinking water’s extremely low pH levels (ranging from 3.18 to 3.82) caused by ProPhorce™ SA Exclusive and Premium acid. 

ProPhorce™ SA Exclusive and Premium acid did not significantly affect the pH of the birds’ crop contents, likely due to the feed’s buffering ability [20]. This could also explain why gross lesions occurred only in the oral cavity and the upper esophageal area, whereas no lesions were seen in the crop and the lower part of the esophagus. Finally, despite the fact that the drinking water was so acidic in terms of inducing gross lesions in birds, it is unclear that the water consumption was not extremely reduced in these animal groups. However, for birds, it is reported that the taste glands are primarily distributed in the epithelium of the upper beak (palate) and to a lesser extent in the region of the anterior tongue, where they are mainly found in other animals [69]. Thus, when a bird can taste something, it is too late to change its response about swallowing [64,70]. 

Neither gross lesions nor extreme reduction in the water pH were noted for the Salgard^®^ Liquid, which is an acidic blend based on organic acid salts (ammonium formate and propionate). Several advantages have been reported for the salts of organic acids compared to their free acid forms. Specifically, organic acid salts are easier to handle, less corrosive to processing equipment, and odourless to the birds. In addition, the free organic acids can be absorbed in the upper gastrointestinal tract or buffered in the acidic pH of the stomach; instead, their salt forms can effectively bypass into the intestine and they can be subsequently converted into their free forms in the lower parts of the gastrointestinal tract where colonization by bacterial pathogens, such *C. jejuni*, usually takes place [71]. However, an essential disadvantage of the salt forms is that they do not reduce the pH value to the same extent as the free acids due to the replacement of the H+ ion by other cations, such as NH^+^, as in the case of salts of the Salgard^®^ Liquid in our study. Ragaa et al. [72] reported that adding an acidifier blend of free organic acids and salts significantly reduced *Clostridium* spp. counts in the intestine. In the present study, the continuous application of Salgard^®^ Liquid reduced *Clostridium* spp. counts in the ceca of birds.

Individual organic acids or blends of organic acids have shown promising anti-*Campylobacter* activity in previous in vitro studies; however, only a few data are available for the anti-*Campylobacter* activity of commercially available formulas. Mantzios et al. [10] reported that Premium acid at a concentration of 0.07% *v*/*v* efficiently inhibited the growth of tested *Campylobacter* spp. strains, whereas at higher concentrations (0.142% *v*/*v*), the product efficiently inhibited the growth of other important zoonotic bacteria such as *S*. Typhimurium, *Listeria* spp., *E. coli*, and *S. aureus*. Likewise, the antimicrobial activity of ProPhorce™ SA Exclusive in vitro has been previously highlighted by Kovada et al. [73] who reported that the product was efficient in inhibiting the growth of tested *C. jejuni* strains in concentrations of 700–1000 mg/Lt (0.07–0.10% *v*/*v*), whereas higher concentrations of the product were required for the inhibition of the tested *E. coli* and *S.* Typhimurium strains 2000–2200 mg/Lt (0.20–0.22% *v*/*v*). For Salgard^®^ Liquid, no previous data are available regarding its antibacterial activity in vitro; however, individual compounds, such as ammonium formate and formic acid, which are highly concentrated in the products, were previously described as efficient anti-*Campylobacter* agents [16,73]. 

Despite the in vitro potential of the tested products, none of these have prevented the transmission of *C. jejuni* from the seeder to sentinel birds in the present work. In all the challenged groups, the transmission rate of *C. jejuni* from seeder to sentinel birds was 100% seven days post-infection, which concurs with the findings of previous investigations [74]. The exact route of transmission is yet to be determined. Litter, feed, or drinking water contaminated by faeces are only a few of the possible routes that may be involved in the transmission of *C. jejuni* to sentinel birds in the challenged groups of this study. It is reported that a single dose of 40 CFU-viable *C. jejuni* can effectively colonize the chicken intestinal tract [39]. 

Although water acidification may not be the golden standard to prevent *C. jejuni* transmission among birds, reducing the *C. jejuni* counts in their gastrointestinal tracts can still be helpful in promoting the One Health approach. In particular, it is reported that reducing *C. jejuni* counts in the ceca of broilers by 3 log_10_ could reduce the incidence of human infection attributed to broiler meat consumption by 58% [13]. In our case, all the products revealed a potential anti-*Campylobacter* activity by reducing the *C. jejuni* counts in birds’ gastrointestinal tracts by 0.15 to 1.51 log_10_ CFU/g depending on the product and the examined anatomical part. 

For *C. jejuni*, the optimum pH range for growth is 6.5–7.5, whereas bacterial cells are killed at a pH under 2.3 [75]. It is important here to note that even when the drinking water in some treatments had a pH of 3.0–3.5, the crop’s content’s pH was not significantly affected, indicating the high buffering capacity of the feed [76]. Moreover, even when the pH in the crop is significantly reduced, it has been reported by previous investigators that despite their high susceptibility, *Campylobacters* have been observed to recover effectively within 24 h, even after being exposed to pH values as low as 3.8 [75]. These facts could explain why the *C. jejuni* counts in the crop of the birds in some groups were not significantly affected (Table 11), even when, in this anatomical part, the tested products are less modified. 

Free organic acids such as those included in the tested commercial formulas of the acidifiers can be readily absorbed by the upper gastrointestinal tract of birds and incorporated into the muscles [20,21], thus, not being in high-enough concentrations to reduce *C. jejuni* counts in the ceca of birds. To that end, novel formulas for acidifiers are being designed with, e.g., with esterification and/or microencapsulation, to ensure the safe bypass of the ingredients through the stomach’s acidic environment and to exert their antimicrobial activities in the intestines of birds [77,78]. 

Finally, organic acids and their derivates could be used in poultry feed or water as antimicrobial agents, preservatives, pH regulators, antioxidants, and flavouring agents. However, according to the category used, organic acids must comply with the maximum levels established by the EU legislation and be authorized by the EU Commission. The use of organic acids as feed additives for poultry is regulated by EU Regulation 1831/2003. It is important to note that organic and inorganic acids are not antibiotics. However, accompanied by excellent nutrition, management, and biosecurity procedures, they can help poultry to maintain intestinal health, as well as to enhance the livability and final productivity of the broilers, strengthen the immune system, improve gut microbiota composition, and, finally, combat intestinal diseases [20,23,78].

The results of the present study are only indicative of the activity of products classified as acidifiers in poultry nutrition. However, it is important to note that the efficacy of commercial formulas in this field can be affected by several factors described above [20]. Therefore, the farmers need to consult a veterinarian or nutritionist in order to design the appropriate, for each farm, dosage scheme.

## 5. Conclusions

Our results demonstrated that continuous acidification of the drinking water did not affect the transmission of *C. jejuni* from the seeder to sentinel birds under the regimens used in this study. In testing the commercial acidifiers at the maximum dosages recommended by the manufacturers, there was a reduction of the *C. jejuni* counts in the gastrointestinal tract of broilers by 0.15 to 1.51 log_10_ CFU/g, depending on the product and the examined anatomical part. In addition, all the products effectively ameliorated the alterations in the pH induced by *C. jejuni* infection in the ceca of birds. However, the continuous use of certain products from the first days of life evoked undesirable effects on broilers’ performance, leading to the need to modify the dosage scheme in future investigations. Thus, it can be concluded that despite the in vitro potential of commercial water acidifiers, it may not be achievable to reach, in vivo, the required antimicrobial concentrations in the ceca of birds, as the drinking water would probably be unpalatable for birds. Further in vivo and in-field studies are suggested to be performed with a dose-response experimental design in order to identify an effective treatment scheme for the control of zoonotic agents without imposing restrictions on poultry health and productivity as well as an economic burden on the farming system. 

## Figures and Tables

**Table 1:** Information (synthesis, manufacturer’s recommendations) about the commercial products that were used in this study.

Product	Manufacturer	Synthesis	M.r. Dose ^1^
SPECTRON^®^	HIPRA, Girona, Spain	Enrofloxacin (100 mg/mL)	10 mg/kg BW
ProPhorce™ SA Exclusive	Perstorp Holding AB, Malmö, Sweden	Formic acid (50–60%), Sodium formate (20–30%), L-(+)-lactic acid (5–10%), Cinnamaldehyde (1–5%)	0.2% *v*/*v*
Premium acid	Kanters, Lieshout, The Netherlands	Formic acid (29%), propionic acid (7.5%), lactic acid (9.2%), acetic acid (2.5%), sorbic acid (0.2%), oligofructose syrup (1%), copper sulphate pentahydrate (0.5%), sodium chloride (0.1%), zinc chelate of glycine hydrated (0.1%)	0.2% *v*/*v*
Salgard^®^ Liquid	Anpario plc, Nottinghamshire, UK	Ammonium formate (20%), propionic acid (5.2%), ammonium propionate (1%) and carrier	0.2% *v*/*v*

^1^ M.r. dose: Maximum recommended dosage by the manufacturer (when animals are present). According to the instructions, in some products the dose may be increased, whereas the evaluation of the optimal farm-specific dose is highly encouraged. For the products for which data were available, we included the percentage of the concentrations of individual compounds. It is important to note that all the products may contain additional proprietary ingredients used for stabilization and enhancing effectiveness.

**Table 2:** Effect of drinking water acidification on the mean body weight ($\bar{x}$ ± SEM) of broiler chicks, experimentally challenged by *C. jejuni*.

Period	Experimental Groups						*p* Value
	A	B	C	D	E	F	
1st d *	47.14 ± 0.39			47.58 ± 0.52	46.33 ± 0.53	48.33 ± 0.68	0.139
9th d *	216.11 ± 2.13			210.00 ± 3.70	205.66 ± 3.48	211.20 ± 3.68	0.216
15th d *	433.64 ± 3.74 ^b^			415.75 ± 6.10 ^a^	412.94 ± 5.27 ^a^	421.31 ± 7.43 ^a,b^	0.011
20th d	853.17 ± 16.73 ^b^	828.25 ± 16.42 ^a,b^	830.33 ± 15.24 ^a,b^	796.67 ± 13.66 ^a^	791.17 ± 12.66 ^a^	837.67 ± 17.72 ^a,b^	0.039
23rd d	1018.08 ± 21.17 ^b^	980.08 ± 19.27 ^a,b^	984.33 ± 18.63 ^a,b^	929.83 ± 17.49 ^a^	933.33 ± 14.40 ^a^	998.83 ± 21.17 ^b^	0.005
25th d	1148.92 ± 24.58 ^b^	1088.92 ± 21.87 ^a,b^	1098.42 ± 20.84 ^a,b^	1042.17 ± 19.93 ^a^	1035.50 ± 16.72 ^a^	1114.50 ± 23.98 ^b^	0.002
30th d	1585.50 ± 47.70	1545.67 ± 42.93	1533.17 ± 47.93	1440.83 ± 40.99	1443.00 ± 36.81	1563.17 ± 49.34	0.100
37th d	2381.17 ± 75.68	2293.83 ± 83.64	2222.67 ± 72.29	2129.83 ± 64.32	2189.83 ± 61.06	2316.50 ± 85.08	0.192
39th d	2634.67 ± 82.70 ^b#^	2532.67 ± 99.21 ^a,b#^	2462.67 ± 87.33 ^a,b#^	2290.00 ± 50.29 ^a#^	2439.50 ± 75.36 ^a,b#^	2558.67 ± 94.73 ^b#^	0.084

^a,b^ Means in the same row with a different superscript differ significantly (*p* ≤ 0.05). ^#^ Means in the same row with a different superscript differ significantly (*p* ≤ 0.10). * For the period 1–18 days, groups A, B, and C were considered as one (controls), as birds in these groups received only tap water (without any treatment), and the challenge by *C. jejuni* was not yet applied.

**Table 4:** Chemical analysis of the drinking water.

Analysis	Value
pH	7.58
Conductivity (μS/cm)	815
Chlorine Cl_2_ (mg/L) (free/total)	0.1/0
Chlorine ions CL^−^ (mg/L)	32.5
NH_4_^+^ (mg/L)	0.034
NO_3_^−^ (mg/L)	9.98
NO_2_^−^ (mg/L)	0.024
Total hardness (mg/L CaCO_3_)	14.37
Fe (mg/L)	0.05
(PO_4_)^3−^ (mg/L)	0.39
(SO_4_)^2−^ (mg/L)	11

**Table 5:** Effect of the tested acidifiers, on the pH ($\bar{x}$ ± SEM) of the drinking water.

Product Dosage	Experimental Groups						*p* Value
	A	B	C	D	E	F	
Low (0.1% *v*/*v*)	7.60 ± 0.01 ^d^	7.60 ± 0.01 ^d^	7.60 ± 0.01 ^d^	3.82 ± 0.02 ^b^	3.72 ± 0.03 ^a^	6.47 ± 0.04 ^c^	<0.001
High (0.2% *v*/*v*)	7.57 ± 0.02 ^d^	7.57 ± 0.02 ^d^	8.17 ± 0.04 ^e^	3.59 ± 0.02 ^b^	3.18 ± 0.04 ^a^	5.53 ± 0.03 ^c^	<0.001

^a,b,c,d,e^ Means in the same row with a different superscript differ significantly (*p* ≤ 0.05).

**Table 6:** Effect of drinking water acidification on the mean water intake (WI) and its ratio with feed intake (WI/FI) ($\bar{x}$ ± SEM) of broiler chicks experimentally challenged by *C. jejuni*.

Parameter	Period	Experimental Groups					
		A	B	C	D	E	F
Water intake/bird (mL)	1–9 d *	74.00			72.93	72.99	81.65
	1–15 d *	100.00			102.05	99.80	109.78
	15–39 d	258.68	260.18	260.07	285.01	287.41	300.58
	1–39 d	177.27	163.14	163.07	178.48	178.28	189.30
Water intake/Average daily feed intake ratio	1–9 d *	2.80			2.74	2.75	3.19
	1–15 d *	2.40			2.66	2.60	2.94
	15–39 d	1.93	2.05	2.01	2.29	2.27	2.33
	1–39 d	2.04	2.03	2.00	2.28	2.26	2.38

Water intake was not evaluated statistically due to the absence of statistical replicates. * For the period 1–18 days, groups A, B, and C were considered as one (controls), as birds in these groups received only tap water (without any treatment), and the challenge by *C. jejuni* was not yet applied.

**Table 7:** Effect of drinking water acidification on the dysbacteriosis lesion score (scale: 0–10) of broiler chicks experimentally challenged by *C. jejuni*.

Score	Experimental Groups						X^2^ Value
	A	B	C	D	E	F	
0	8.33%	8.33%	0%	16.67%	8.33%	8.33%	0.406
1	8.33%	33.33%	25%	50%	25%	33.33%	
2	58.33%	25%	33.33%	8.33%	41.67%	41.67%	
3	25%	33.33%	25%	8.33%	25%	16.67%	
4	0%	0%	16.67%	16.67%	0%	0%	
Total	100%	100%	100%	100%	100%	100%	

During this lesion score, none of the birds were given a score of 5, 6, 7, 8, 9, or 10. At the 39th day of age, dysbacteriosis lesion scores were not significantly (*p* > 0.05) different according to the chi-2 analysis, and, therefore, multiple comparisons with Kruskal–Wallis test were not performed.

**Table 8:** Effect of drinking water acidification on the occurrence of gross lesions (erosions or/and ulcers) in the upper gastrointestinal tract (oral cavity and esophagus) of birds.

	Experimental Groups						X^2^ Value
	A	B	C	D	E	F	
No lesions	90.90%			37.5%	50%	87.5%	
Lesions	9.10%			62.5%	50%	12.5%	
Total	100%			100%	100%	100%	0.005

For the period 1–18 days, groups A, B, and C were considered as one (controls), as birds in these groups received only tap water (without any treatment), and the challenge by *C. jejuni* was not yet applied. Kruskal–Wallis’ test: controls vs. group D: *p* = 0.022; controls vs. group E: *p* = 0.020; group D vs. group F: *p* = 0.022.

**Table 9:** Effect of drinking water acidification on the mean pH ($\bar{x}$ ± SEM) of the contents in the various anatomical parts of the gastrointestinal tract of broiler chicks, experimentally challenged by *C. jejuni*.

Anatomical Part	Experimental Groups						*p* Value
	A	B	C	D	E	F	
*15th day* *							
Crop	4.77 ± 0.08			4.98 ± 0.19	4.84 ± 0.09	4.89 ± 0.16	0.642
Gizzard	2.88 ± 0.10			2.79 ± 0.14	2.45 ± 0.16	2.62 ± 0.21	0.154
Duodenum	6.16 ± 0.05			6.24 ± 0.03	6.17 ± 0.06	6.28 ± 0.07	0.563
Jejunum	5.92 ± 0.04			5.98 ± 0.05	5.76 ± 0.04	5.91 ± 0.05	0.086
Ileum	5.42 ± 0.15 ^a^			6.34 ± 0.20 ^b^	5.59 ± 0.15 ^a^	5.56 ± 0.21 ^a^	0.015
Ceca	6.26 ± 0.09			6.19 ± 0.15	5.84 ± 0.15	6.05 ± 0.16	0.131
*39th day*							
Crop	4.93 ± 0.33	4.54 ± 0.20	4.87 ± 0.14	4.60 ± 0.26	4.84 ± 0.12	4.49 ± 0.08	0.524
Duodenum	6.08 ± 0.03	6.06 ± 0.05	6.06 ± 0.06	6.09 ± 0.08	6.03 ± 0.06	6.23 ± 0.05	0.143
Jejunum	6.57 ± 0.13	6.68 ± 0.13	6.60 ± 0.15	6.62 ± 0.15	6.76 ± 0.08	6.36 ± 0.11	0.350
Ileum	7.43 ± 0.15	7.78 ± 0.19	7.71 ± 0.23	7.66 ± 0.20	7.83 ± 0.14	7.19 ± 0.25	0.219
Ceca	6.37 ± 0.11 ^a^	6.94 ± 0.06 ^b^	6.16 ± 0.10 ^a^	6.52 ± 0.08 ^a^	6.37 ± 0.19 ^a^	6.31 ± 0.14 ^a^	0.008

^a,b^ Means in the same row with a different superscript differ significantly (*p* ≤ 0.05). * For the period 1–18 days, groups A, B, and C were considered as one (controls), as birds in these groups received only tap water (without any treatment), and the challenge by *C. jejuni* was not yet applied.

**Table 10:** Effect of drinking water acidification on the mean viscosity ($\bar{x}$ ± SEM) (cP) of the intestinal contents of broiler chicks, experimentally challenged by *C. jejuni*.

Anatomical Part	Experimental Groups						*p* Value
	A	B	C	D	E	F	
*15th day* *							
Jejunum	1.39 ± 0.04			1.60 ± 0.18	1.43 ± 0.05	1.62 ± 0.05	0.119
Ileum	1.69 ± 0.15			- ^1^	1.82 ± 0.19	1.67 ± 0.04	0.862
*39th day*							
Jejunum	3.24 ± 0.25 ^a,b,c^	2.43 ± 0.15 ^a^	2.85 ± 0.14 ^a,b,c^	2.77 ± 0.17 ^a,b^	3.44 ± 0.27 ^c^	3.29 ± 0.29 ^b,c^	0.021
Ileum	3.16 ± 0.10	3.47 ± 0.20	3.35 ± 0.09	3.38 ± 0.12	3.50 ± 0.18	3.46 ± 0.19	0.761

^a,b,c^ Means in the same row with a different superscript differ significantly (*p* ≤ 0.05). * For the period 1–18 days, groups A, B, and C were considered as one (controls), as birds in these groups received only tap water (without any treatment), and the challenge by *C. jejuni* was not yet applied. ^1^ These data are not available due to insufficient digesta for measuring the viscosity in the ileum of birds in this group.

**Table 11:** Effect of drinking water acidification on the mean *C. jejuni* counts (log_10_ $\bar{x}$ ± SEM) in the crop and ceca of broiler chicks, experimentally challenged by *C. jejuni*.

Anatomical Part	Experimental Groups							*p* Value
	A	B	C	D	E	F	A	
		*25th day of age*						
Crop	ND	5.29 ± 0.50 ^b^		3.79 ± 0.28 ^a^	4.19 ± 0.36 ^a^	3.78 ± 0.22 ^a^	4.55 ± 0.33 ^a,b^	0.020
Caecum	ND	7.09 ± 0.33 ^c^		5.61 ± 0.17 ^a^	6.54 ± 0.22 ^b,c^	6.70 ± 0.18 ^b,c^	6.39 ± 0.18 ^b^	0.001
		*39th day of age*						
Crop	ND	6.68 ± 0.12		6.52 ± 0.15	7.13 ± 0.17	7.08 ± 0.12	6.53 ± 0.34	0.096
Caecum	ND	7.99 ± 0.15 ^b^		7.39 ± 0.18 ^a^	7.68 ± 0.21 ^a,b^	7.31 ± 0.08 ^a^	7.84 ± 0.20 ^a,b^	0.040

^a,b,c^ Means in the same row with a different superscript differ significantly (*p* ≤ 0.05). ND: not detected.

**Table 12:** Effect of drinking water acidification on the mean *E. coli*, *Clostridium* spp., *Lactobacilli*, and *Bifidobacterium* counts (log $\bar{x}$ ± SEM) in the ceca of broiler chicks experimentally challenged by *C. jejuni*.

Tested Bacteria	Experimental Groups						*p* Value
	A	B	C	D	E	F	
15th day *							
*E. coli*	5.08 ± 0.35			4.86 ± 0.42	4.75 ± 0.19	5.08 ± 0.73	0.951
*Clostridium* spp.	4.70 ± 0.25 ^b#^			4.80 ± 0.32 ^b#^	4.15 ± 0.42 ^b#^	3.50 ± 0.29 ^a#^	0.073
*Lactobacilli*	7.03 ± 0.20			6.96 ± 0.23	6.82 ± 0.48	6.42 ± 0.32	0.525
*Bifidobacterium*	6.53 ± 0.14			6.02 ± 0.21	6.43 ± 0.36	6.24 ± 0.09	0.347
39th day							
*E. coli*	5.45 ± 0.15	4.96 ± 0.48	5.29 ± 0.29	5.25 ± 0.40	5.96 ± 0.61	5.16 ± 1.16	0.891
*Clostridium* spp.	4.00 ± 0.00 ^a^	5.70 ± 0.00 ^b^	5.63 ± 0.27 ^b^	5.78 ± 0.07 ^b^	5.12 ± 0.52 ^b^	5.72 ± 0.19 ^b^	0.014
*Lactobacilli*	7.04 ± 0.74	6.63 ± 0.33	7.38 ± 0.24	7.35 ± 0.35	7.38 ± 0.15	6.45 ± 0.15	0.425
*Bifidobacterium*	7.03 ± 0.42	6.73 ± 0.31	6.35 ± 0.35	6.48 ± 0.30	6.60 ± 0.30	5.90 ± 0.45	0.442

^a,b^ Means in the same row with a different superscript differ significantly (*p* ≤ 0.05). ^#^ Means in the same row with a different superscript differ significantly (*p* ≤ 0.10). * For the period 1–18 days, groups A, B, and C were considered as one (controls), as birds in these groups received only tap water (without any treatment), and the challenge by *C. jejuni* was not yet applied.

## Data Availability

None of the data presented were deposited in an official repository.

## Supplementary Materials

The following supporting information can be downloaded at: https://www.mdpi.com/article/10.3390/ani13122037/s1, Table S1. Ingredients and calculated analysis of the starter (1 to 15 days) and for finisher (16 to 39 d) diets. Table S2. Composition of reagents used for *C. jejuni* DNA extraction.
